# What the Danish Women Learn

**Published:** 1891-06

**Authors:** 


### WHAT THE DANISH WOMEN LEARN.

The Danish girl is little known and less understood in foreign countries. Even the commonplace German girl is much more frequently described and analyzed than she, in the modern literature of foreign countries. She is, however, worthy of all the observation which has been so freely bestowed of late years on her continental sisters. For Canadians especially her training and ideas of a girl's duties in life should be subjects of keen interest as she grows to womanhood with more Canadian characteristics than almost any other girl between the Bay of Biscay and the Asiatic border.

The Danish girl in her youth does not learn so much soldier-like obedience and reverence as the German girl, nor so much imitation of knowledge as the American and English girls. The object of the education of young girls in Denmark is to render them really independent for life. Every effort is made to develop the reasoning powers and to arouse originality. "Keys" and "ponies" are consequently almost unknown among Danish schoolgirls. A term in metaphysics just because it smacks of higher education, and gives an opportunity of saying, "Oh, how I hated it!" is an unknown institution. Everything, on the contrary, is careful, solid, thorough.

Among the very young, object lessons are more prevalent than in any other country. Among the more mature the same principle obtains, and all the natural sciences, for instance, are taught with the aid of sufficient apparatus, specimens and charts. The Danish girl gets the same manual training as the boys with whom she is allowed to associate in school hours.

The consequence of this early training is that most Danish girls leave school with all sorts of un-European ideas of their independence. Probably no continental country has, in proportion to its size, as many women graduates in medicine, philology and jurisprudence, and the number of girls in professional and learned courses of study is increasing yearly. Daughters of rich tradespeople devote themselves to the trades of their apprenticeships in the concerns of strangers, where only the value of their services determines the rapidity of their advancement.

The trade of the goldsmith has within the last fifteen years gained popularity steadily among daughters of the better class of Danes, so that to-day all the goldsmiths of Copenhagen have young women as apprentices as well as assistants often enough in their most elaborate styles of work. Young women of lower aspirations become bakers or confectioners, and are slowly but surely crowding out from the trades the men to whom such occupations never properly belonged.

Not a few girls in Denmark study the art of gardening. Scores annually become professional dairymaids. Young women of the peasant class devote themselves mostly to learning all about vegetable gardening, and to encourage them in learning to do their work thoroughly, numerous agricultural associations and local authorities give prizes for the greatest skill shown in transforming ugly and unfruitful spots into model beds of beets, peas, and the like.

"High and low, rich and poor," said a German educator, in a recent article on Danish women, "teach their daughters to support themselves in case of need without being obliged to stifle their lives in the crowded rooms of great factories and workshops."

---

### HINDU WOMEN.

An Englishman's first idea is to ask his friend to dinner, his next to make the acquaintance of his wife and daughters. With a Hindu you can do none of these things. It is often better not to refer to them.

A Mahommedan will dine with you, but his ladies, with few exceptions, are even more jealously secluded than those of the Hindu. Nor do the women, for the most part, seem to desire more liberty. Many of them know very well how to manage their husbands, and if they want to go any where, or to see any thing, the men have to find some means of gratifying them. The reverence paid to mothers is extreme. I know a man of high position and of middle age who is obliged to worship gods in whom he does not believe, for fear of displeasing his mother, and another who cannot make the pilgrimage which he desires to Benares, because custom would oblige him to take his mother on his first visit to the holy city, and she is unfit to travel. But most Indian women are too uneducated to take pleasure in mixing in a society whose ways and thoughts are totally different from their own. Efforts are being made to teach them, and there is little doubt that when they know a good deal about the world they will wish to see it, and that when this becomes their object they will speedily attain it. Certainly it will be better to fit them for a position before calling upon them to occupy it. A somewhat similar remark applies to infant marriages and child widows. The women must desire change before it is made. A philanthropic maiden lady, who had passed her first youth, was conversing not long ago with a married Indian lady and her widowed sister-in-law on these topics. After she left them the married lady said, "I married at seven, and my husband was nine years old. We have lived happily together. How is it that this lady has not married till her hair is growing gray? Has nobody asked for her! There ought to be a law in England that no one shall remain unmarried after a certain age." The comment of the sister-in-law on the attack made upon her was simply, "Why does not the Empress marry again?"—*The Nineteenth Century.*

